# First aid skill retention of first responders within the workplace

**DOI:** 10.1186/1757-7241-19-11

**Published:** 2011-02-08

**Authors:** Gregory S Anderson, Michael Gaetz, Jeff Masse

**Affiliations:** 1 Kinesiology and Physical Education, University of the Fraser Valley, 33844 King Rd., Abbotsford, BC Canada

## Abstract

**Background:**

Recent literature states that many necessary skills of CPR and first aid are forgotten shortly after certification. The purpose of this study was to determine the skill and knowledge decay in first aid in those who are paid to respond to emergency situations within a workplace.

**Methods:**

Using a choking victim scenario, the sequence and accuracy of events were observed and recorded in 257 participants paid to act as first responders in large industrial or service industry settings. A multiple choice exam was also written to determine knowledge retention.

**Results:**

First aid knowledge was higher in those who were trained at a higher level, and did not significantly decline over time. Those who had renewed their certificate one or more times performed better than those who had learned the information only once. During the choking scenario many skills were performed poorly, regardless of days since last training, such as hand placement and abdominal thrusts. Compressions following the victim becoming unconscious also showed classic signs of skill deterioration after 30 days.

**Conclusions:**

As many skills deteriorate rapidly over the course of the first 90 days, changing frequency of certification is not necessarily the most obvious choice to increase retention of skill and knowledge. Alternatively, methods of regularly "refreshing" a skill should be explored that could be delivered at a high frequency - such as every 90 days.

## Background

Unintentional injuries are the leading cause of death among persons 1-34 years of age in Canada [1] and 1-44 years in the United States, resulting in approximately 2.6 million hospitalizations, 34.9 million emergency room visits and 87.6 million medical office visits per year for all workers in the U.S. [2]. Basic first aid training prepares bystanders to react and provide immediate and efficient treatment for a wide variety of incidents including alerting the emergency medical system (EMS), maintaining the airway, breathing and circulation, respiratory and cardiac arrest, and hemorrhage control. The response time in emergency situations is critical, but the first aid provided must be performed properly in order to prevent further complications and potentially save lives [3].

To improve the emergency response and outcome, first aid must be taught correctly to a broad spectrum of individuals within the community, workplace, and health care environment. However, with the need for effective initiation of intervention being known, healthcare professionals and laypersons often face criticism for inadequate basic lifesaving skills [3-5]. Insufficient skills of basic lifesaving are caused by a lack of training and appropriate instruction, limited practice, lack of self-efficacy, and poor skill retention [4]. While millions of people are being trained each year, the efficacy of this training, and the subsequent performance of the skills learned, has come into question [6,7]. Current literature states that many necessary skills of first aid are forgotten shortly after certification with rapid deterioration of skills and knowledge in two to six months [8-12]. As there is an expectation that immediate and effective emergency life-saving procedures will be provided within the workplace by trained personnel, the purpose of this study was to examine the extent to which first aid skills are retained in an industrial or service oriented workplace environment.

## Methods

Following institutional ethical review board approval, participants were recruited by contacting large industrial employers in the Greater Vancouver area of British Columbia, Canada that regularly contracts out first aid training. All employees that were paid to provide first aid within the workplace were eligible to participate. Researchers approached the person/people in charge of safety and/or first aid to gain permission and recruit participants.

All data were collected at the worksite by a trained first aid instructor who had training as an examiner of practical skills. Employers were instructed to schedule their employees to participate in the study individually throughout their day and give them no details except that it was a first aid study. Participants entered the study room individually, where they met the researchers and gained their first knowledge of the details of the study. After their demographic information was recorded, participants were introduced to the first aid scenario set up in an adjacent room.

The choking scenario (an unresponsive foreign body airway obstructed victim) involved a training manikin (Resusci^®^Anne SkillReporter; Laerdal, Stavanger, Norway) in a wheelchair that was choking at a restaurant. The participant was told "you are in a restaurant, you see a person in a wheelchair grabbing their throat and attempting to cough. The person is wheelchair bound. The person is unable to cough. No one knows what happened. Everything is as found unless we tell you otherwise." A timestamp program using a Microsoft EXCEL spread sheet was used to record and assess the correct order and proper execution of each of the steps for the first aid scenario. The Laerdal recording manikin was connected to sensors and a computer that recorded the rate, depth, and frequency of breathing, and the rate, depth, and location of chest compressions. The participants were observed for proper scene safety and management of the airway, which included abdominal thrusts to clear the airway. The participants were told the victim lost consciousness after one minute had elapsed and the victim was then lowered to the floor by the participant. Alerting EMS, managing the airway, providing ventilations, performing compressions to clear the airway, and monitoring of ABC's were evaluated after moving the victim to the floor.

Participants were told they could ask questions of the researchers, and standard answers were given for common questions asked. When asked to phone 911, the researchers replied with "I can do that", and then they told them "EMS will be here in about 10 minutes." When asked any specific question that could bias the outcome of the scenario the reply was to "do what you would do in real life." The participants were told that the manikin was wheelchair bound and could not be moved from the wheelchair until it lost consciousness (after the first minute of the choking scenario) because of the difficulty in repeatedly standing the mannequin in a self-supportive manner, and in order to discern whether participants knew the proper protocol for clearing the airway of a person in a wheelchair.

After completing the scenario, participants completed a written multiple choice first aid exam that used questions from the Worker's Compensation Board (WCB) of British Columbia's first aid exam. Participants that had completed level one certification answered the first 10 questions of the exam, level two participants completed questions 1-15, and participants with level three certifications completed all 20 questions in the exam booklet. Level 1 first aid training encompasses 8 hours and trains candidates to recognize and intervene in life-threatening conditions in the workplace. The roles and responsibilities of the occupational first aid attendant, human anatomy and physiology, ABC interventions, and minor wound care are discussed and practiced. Level 2 training provides 16 hours of comprehensive training in first aid for the workplace. Candidates learn the same priorities of emergency care used by health care professionals in the pre-hospital setting and expand level 1 training by adding content in injuries due to heat and cold, bone and joint injuries, spinal injuries, specific medical conditions, minor wound care, and poisons. Level 3 training spans 70 hours and integrates the latest medical assessments, techniques and interventions using a variety of safety devices and techniques for emergency situations and rescue. At each level participants were required to obtain 70% on each of the written, oral and practical portions of the examination process in order to be certified.

### Data Assembly

Data were assembled in a series of Microsoft EXCEL spread sheets. Descriptive statistics were calculated using EXCEL functions. Descriptive and graphical data are reported based upon the groupings of "days since last training". In each case data were assembled using the following categories: category 1 = 1-30 days (<1 month); 2 = 31-90 days (1 - 2.9 months); 3 = 91 - 182 days (3 - 5.9 months); 4 = 183 - 364 days (6 - 11.9 months); 5 = 365 - 546 days (12 - 17.9 months); 6 = 547 - 729 days (18 - 23.9 months); 7 = 730 - 1094 days (24 - 35.9 months); and 8 = >1094 days (3 or more years).

### Data Analysis

Both descriptive and quantitative analyses were performed. Independent samples t-tests with Levene's test for equality of variances were used to assess the effect of re-certifications (zero versus one or more) on test scores. Effect sizes (*d*) were calculated based on Cohen [13]. Regression analyses were performed to explore the impact of the number of days since the person was last trained, and to investigate the relationship between previous training and recertification on performance measures using SPSS version 10.

### Limitations

Participants in this study were trained, while primarily by one service provider, by various instructors. Further, each participant was faced with an artificial situation which may, in some instances, impact performance times. Skilled performance on a manikin may also be difficult, as some have problems locating the correct anatomical landmarks.

## Results

A total of 257 participants had first aid training and complete data. Of these, 154 were male, and 103 were female, with an average age of 34.0 years. The distribution of participants across the 8 categories of "days since training" described in the methods are provided in Table 1.

**Table 1:** Distribution of participants across categories of "days since training"

Category	Days Since Training	Number (n)	Male (n)	Female (n)	Average Age (yr)
1	1 - 30	21	11	10	30.5
2	31 - 90	34	26	8	36.2
3	91 - 182	41	23	18	33.2
4	183 - 364	48	29	19	33.5
5	365 - 546	43	24	19	35.1
6	547 - 729	25	14	11	33.5
7	730 - 1094	18	12	6	32.3
8	>1094	27	15	12	35.3

**Totals**		257	154	103	34.0

The participants in the present study were distributed across 14 different WCB occupation codes, with the largest number in accommodation, food, and leisure services, followed by manufacturing other products, other services, transportation and related services, general construction, utilities and warehousing. The majority of participants in each "days since last training" category had not been re-certified, and held Level 1 certification. In all categories, at least one participant was previously certified at a higher level which they no longer held.

**Figure 1:**
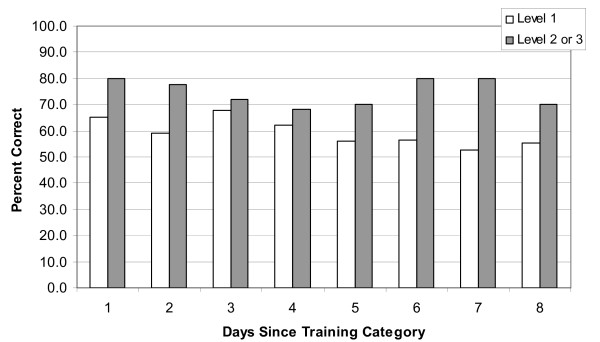
**Average scores on the first aid multiple choice exam (first 10 questions) for those with Level 1 training, and those with greater than Level 1 training**.

**Figure 2:**
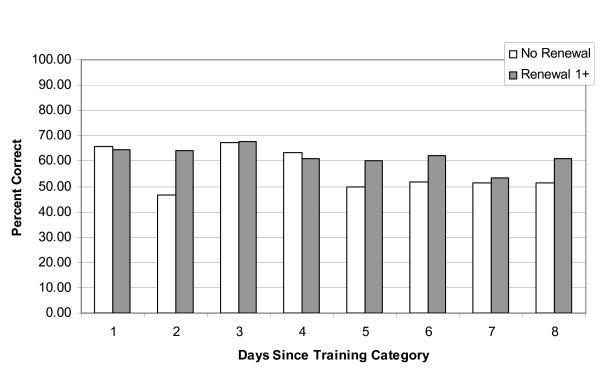
**A comparison of multiple choice exam scores between those who were trained once and those who had renewed their first aid at least once**.

First aid knowledge, calculated using the first 10 questions of the multiple choice exam was higher in those who were trained at a higher level (Figure 1), and did not significantly decline over time. For the independent samples t-tests, Levene's Test for equality of variance was not significant (*F *= 1.579; *p *= .210) and equal variances were assumed. Of those who held a Level 1 first aid certificate, those who had renewed their certificate one or more times performed better than those who had learned the information only once [*t*(252) = 2.61; *p *= 0.01; *d *= 0.34, small-medium effect] (results also shown in Figure 2). The differences between those who had not re-certified versus those who re-certified at least once was most evident for those who had no recent training experience.

The victim for the choking scenario was in a wheel chair, and many individuals were confused on how they would proceed because of this. Once deciding to engage, fewer than 5% of the participants ensured they were in no danger before proceeding with first aid treatment for choking, and only 27% specifically asked the person if they were choking. During the treatment of the victim, 41% of the participants used gloves, and 65% used the pocket mask provided.

The physical skills required to dislodge a foreign body and reinstate unassisted breathing were also not performed well. Only 33% of the participants could correctly perform abdominal thrusts with correct hand placement, and once the victim became unconscious, only 52% activated the EMS. Once unconscious, 45% of the participants correctly placed their hands in order to perform compressions; 74% of the participants performed compressions, although only 31% performed the compressions to the trained standard. The percentage of persons completing each step based on days since training are presented in Table 2.

**Table 2:** Choking scenario: Percentage of person's completing each step based on days since training

	Days Since Training							
	
	1-30	31-90	91-182	183-365	366-547	548-730	731-1095	1096+
**Scene Safety**								
Ensure No Danger	5	0	15	6	2	4	0	4
Gloves	52	41	46	44	42	40	27	38
Pocket Mask	57	68	71	73	65	56	53	50

**Airway**								
Ask "Are you choking"	38	21	34	29	21	32	20	29
Determine if patient can speak or cough	71	79	78	63	58	72	7	38

**Clear the Airway**								
Abdominal thrust - correct placement	14	38	80	31	26	16	20	8
Repeat thrusts (airway cleared/unconscious)	19	21	76	21	21	20	7	0

**EMS**								
Activate EMS	33	62	71	50	49	40	33	52

**Airway**								
Open the airway (appropriate technique)	62	53	59	58	72	36	33	29
Look in mouth for foreign body	57	53	59	60	56	40	53	29

**Breathing**								
Seal pocket mask properly	62	50	71	67	65	28	47	25
Attempt to ventilate	71	53	71	77	70	52	53	58
Reposition the head	67	15	34	38	40	20	7	17
Re-attempt to ventilate	48	47	56	48	53	40	33	29

**Figure 3:**
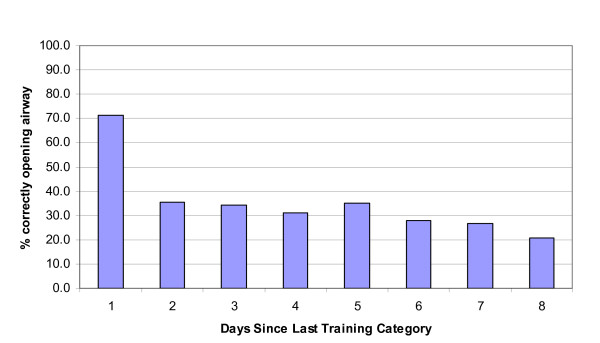
**Percentage of participants in each "days since last training" category who correctly opened the airway**.

**Figure 4:**
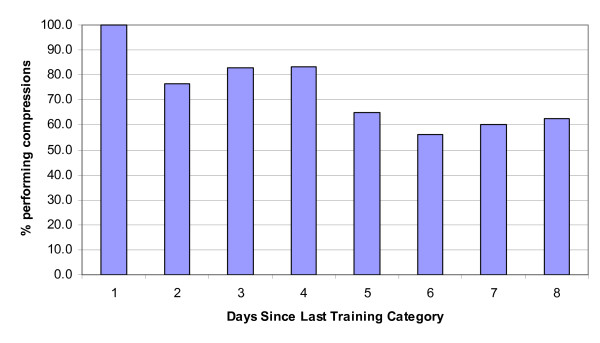
**Percentage of participants in each "days since last training" category who correctly performed compressions after their victim was unconscious**.

Knowledge components of ensuring no danger and remembering to activate the EMS were performed poorly regardless of days since last training. The skill-based components appeared to diminish with time. An abrupt decrease in opening the airway was evident (Figure 3) after only 30 days. Correct hand placement and abdominal thrust were not performed well by those with 1-90 days of training, but showed deterioration from 90 days onwards. Compressions following the victim becoming unconscious also showed classic signs of skill deterioration after 30 days (Figure 4). The knowledge-based items did not show any typical pattern of decay, although some items (ensure no danger) were performed seldomly.

Results of regression analyses suggest that approximately 40% of the variance in first aid multiple choice exam score could be accounted for by the numbers of times certified (R Squared = 0.401). The correlation among these two variables is approximately 0.64 and is significant (*F *[1, 252] = 171.89, *p *< 0.000). The results for the linear regression of days post certification on score demonstrate that days post certification is a moderate predictor of first aid multiple choice exam score, but not as good as the number of times certified. The results suggest that only 26% of the variance in score can be accounted for by the days since certification (R Squared = 0.256). The correlation among these two variables is approximately 0.51 and also significant (*F *[1, 252] = 86.98, *p *< 0.000). The number of times certified appears to be a much better predictor of performance on subsequent tests of first aid knowledge. Due to the moderate to large correlation between these variables, reasonable predictions of test score based on the number of prior certifications can be made.

## Discussion

The ability of trained personnel in an employment setting to deliver basic lifesupporting first aid is paramount to the safety of their employees. With two year renewal dates, the ability of these trained personnel to provide this critical lifesupporting first aid has been questioned [8-10]. Basic life support skills have to be taught, learned and remembered, and most evidence demonstrates rapid skill decay even among professionals in an occupational and health care setting [8,11,12,14,15]. The results of the present study confirm that many first aid skills deteriorate to what may well be considered unacceptable levels prior to recertification, and as early as 30 days post training. It appears that the skill-based components may deteriorate in a more predictable fashion following training, while the reduction in knowledge would be contaminated by the repetition of training in those that had recertified their first aid one or more times.

Both healthcare professionals and laypersons often face criticism for inadequate basic lifesaving skills typically related to lack of training and appropriate instruction, limited time for practice and refinement of skill, lack of self-efficacy, and poor skill retention [3-5,15]. The present study demonstrates mixed results with skill and knowledge retention. Theoretical knowledge, as demonstrated through multiple choice exam scores, declined over time. Level of mastery has been reported to have a significant effect on the rate of CPR skill decline [16] and appears to be similar in first aid retention of theoretical knowledge. However, other knowledge components were performed poorly regardless of level of training or days since last training. For example, fewer than 5% of the participants ensured they were in no danger before proceeding with first aid treatment for choking, and only 27% specifically asked the person if they were choking. During the treatment of the victim, 41% of the participants used gloves, and 65% used the pocket mask provided.

The physical skill-based components appeared to diminish with time. An abrupt decrease in opening the airway was evident after only 30 days. Correct hand placement and abdominal thrust were not performed well by those with 1-90 days of training, but showed deterioration from 90 days onwards. Compressions following the victim becoming unconscious also showed classic signs of skill deterioration after 30 days. It appears that the physical skill-based components may deteriorate in a more predictable fashion following training, while the reduction in knowledge would be contaminated by the repetition or level of training in those that had recertified their first aid one or more times or had a higher level of certification. Several studies demonstrate limited retention of first aid knowledge and rapid deterioration after initial training [11,12,17,18]. Examining the relationship between knowledge and skill performance in emergency medical technicians Brown et al. [19] found accurate knowledge to be related to better performance of chest compression rate and compression to ventilation ratio, although overall performance was poor. Differential rates of skill and knowledge deterioration are often reported allowing some to retain theoretical knowledge although having poor physical skill retention [17]. Moser and Coleman [20] suggest that CPR skills appear to decline at a faster rate than knowledge, with significant decline in CPR skills occurring as early as two weeks post-training. In both intensive care nurses [21] and airline cabin crew [10] theoretical CPR knowledge retention 12 months post-training was high, but there was an inability to meet the standard passing criteria in CPR skill performance.

## Conclusions

The ability of trained personnel in an employment setting to deliver basic life supporting first aid is paramount to the safety of their employees. With two year renewal dates, the ability of these trained personnel to provide adequate critical lifesupporting first aid can be questioned. Recognizing that skills decay rapidly after original training, employers should be encouraging their first-aiders to refresh their knowledge between recertification and refresher courses, as well as offering BLS courses and skills training to all employees as an added safety precaution. Identifying simple and cost effective strategies for updating skills and knowledge may prove to be beneficial and reduce the rate of skill decay in workplace first aid providers.

## Competing interests

The authors declare that they have no competing interests.

## Authors' contributions

GSA and MG obtained funding for the project and drafted the manuscript. GSA oversaw data collection, while JM collected all data, prepared data for analysis, and helped draft portions of the paper. All authors have revised, read and approved the article.
